# Subcellular Localization of Frizzled Receptors, Mediated by Their Cytoplasmic Tails, Regulates Signaling Pathway Specificity

**DOI:** 10.1371/journal.pbio.0020158

**Published:** 2004-07-13

**Authors:** Jun Wu, Thomas J Klein, Marek Mlodzik

**Affiliations:** **1**Brookdale Department of Molecular, Cell, and Developmental Biology, Mount Sinai School of MedicineNew York, New YorkUnited States of America

## Abstract

The Frizzled (Fz; called here Fz1) and Fz2 receptors have distinct signaling specificities activating either the canonical Wnt/β-catenin pathway or Fz/planar cell polarity (PCP) signaling in *Drosophila.* The regulation of signaling specificity remains largely obscure. We show that Fz1 and Fz2 have different subcellular localizations in imaginal disc epithelia, with Fz1 localizing preferentially to apical junctional complexes, and Fz2 being evenly distributed basolaterally. The subcellular localization difference directly contributes to the signaling specificity outcome. Whereas apical localization favors Fz/PCP signaling, it interferes with canonical Wnt/β-catenin signaling. Receptor localization is mediated by sequences in the cytoplasmic tail of Fz2 that appear to block apical accumulation. Based on these data, we propose that subcellular Fz localization, through the association with other membrane proteins, is a critical aspect in regulating the signaling specificity within the Wnt/Fz signaling pathways.

## Abstract 2

Differential subcellular localization of Fz1 and Fz2 receptors contributes to signaling specificity within the Wnt/Fz signaling pathways

## Introduction

Pattern formation in multicellular organisms relies on inductive signaling events. Several evolutionarily conserved ligand–receptor combinations and associated signal transduction pathways are used again and again during development to induce tissue- and cell-type-specific responses. Thus, context-dependent signaling specificity is an important prerequisite for patterning and differentiation. Although for many signaling pathways the flow of information is largely established, the underlying signaling specificity mechanisms remain unclear.

Members of the Frizzled (Fz) family of seven-pass transmembrane proteins act as receptors for the Wnt family of secreted ligands (Bhanot et al. 1996). In most cases, Wnt/Fz signal transduction leads to posttranslational stabilization of the intracellular protein β-catenin (β-cat) (β-cat or Armadillo [Arm] in *Drosophila*; reviewed in Polakis 1999, 2000). However, recent work has established that some Wnt ligands and Fz receptors can also signal through pathways independent of the Wnt/β-cat (Wg/Arm) cascade in certain contexts in vertebrates and invertebrates (reviewed in Mlodzik 2002; Veeman et al. 2003). In particular, the Fz/planar cell polarity (PCP) pathway has been studied extensively in both *Drosophila* and vertebrates (Adler 2002; Keller 2002; Mlodzik 2002; Tada et al. 2002; Strutt 2003). PCP is easy to study and evident in all adult tissues in *Drosophila*. For example, in wing cells the PCP response is the formation of an actin spike (the wing “hair”) that points distally, and in the eye PCP is manifest in the regular ommatidial arrangement in the anteroposterior and dorsoventral axes (reviewed in Adler 2002; Mlodzik 2002). These distinct PCP manifestations are regulated by the same set of genes, the so-called primary polarity genes, of which Fz is the most prominent and best studied. Similarly, this noncanonical Fz/PCP pathway has been implicated in PCP establishment in vertebrates, with prominent examples including the polarization of the sensory epithelium in the inner ear (Curtin et al. 2003; Dabdoub et al. 2003; Montcouquiol et al. 2003) and aspects of cell polarization in the convergent extension process during gastrulation (for a description of the similarities, pathway conservation, and specific readouts see reviews (Keller 2002; Mlodzik 2002; Veeman et al. 2003). Despite the increasing knowledge about the distinct pathways mediated by Wnt/Fz signaling, the regulation of Fz signaling specificity remains largely obscure.

Both pathways, Wnt/β-cat and Fz/PCP, signal via Disheveled (Dsh) (reviewed in Boutros and Mlodzik 1999). This raises the intriguing question of how structurally very similar receptors can signal through a common protein into distinct downstream effector pathways. In *Drosophila,* Fz (for clarity we will refer to it as Fz1) and Fz2 are functionally redundant receptors for Wg, activating the canonical Wg/Arm cascade (Bhat 1998; Kennerdell and Carthew 1998; Bhanot et al. 1999; Chen and Struhl 1999). In addition to this redundant role in canonical signaling, Fz1 has a specific nonredundant role in the Fz/PCP pathway (Vinson and Adler 1987; Vinson et al. 1989). Subdomains of Fz1 and Fz2 have been analyzed with respect to the functional similarities and differences of the two receptors (Boutros et al. 2000; Rulifson et al. 2000; Strapps and Tomlinson 2001). These studies have suggested that signaling differences between Fz1 and Fz2 could lie in their different affinities for ligands (e.g., Wg has a 10-fold higher affinity for Fz2; Rulifson et al. 2000) and in additional cytoplasmic sequences which govern distinct intrinsic signaling preferences between Fz1 and Fz2 for the canonical and Fz/PCP pathways (Boutros et al. 2000; Strapps and Tomlinson 2001).

Signaling specificity could be regulated by distinct Wnt-Fz combinations that would activate either the canonical or noncanonical pathway. Although a PCP-specific Wnt ligand for Fz1 has not yet been identified in flies, in vertebrates specific Wnt(s)-Fz(s) combinations are associated with either canonical or Fz/PCP signaling. However, the specificity is not simple. For example, although Wnt5a and Wnt11 cause embryonic phenotypes associated with the Fz/PCP-like pathway (Heisenberg et al. 2000; Tada and Smith 2000), coexpression of Wnt5a with Fz5 causes axis duplications, a canonical Wnt/β-cat phenotype (He et al. 1997). Similarly, vertebrate Fz7 receptors have been shown to affect both noncanonical (Djiane et al. 2000; Medina et al. 2000) and β-cat signaling (Kuhl et al. 2000). These data suggest that signaling specificity is not necessarily associated with a particular Wnt ligand or Fz receptor. Wnt/Fz signaling specificity may be determined, in part, by the presence of distinct coreceptors. For example, the Arrow-LRP5/6 protein acts as a Wnt/Wg coreceptor and is only required for Wnt/β-cat signaling (Tamai et al. 2000; Wehrli et al. 2000). No coreceptor of Fz1 has been reported for Fz/PCP signaling. Clearly this is a complicated issue and is likely to be context and cell-type dependent.

Endogenous Fz2 is difficult to detect, but in the wing hinge region it is localized evenly in membranes along the apical–basal axis (M. Strigini, unpublished data). Similarly, overexpressed Fz2 (under *dppGal4* control) is localized throughout the apical–basal axis of larval imaginal disc epithelia, and extracellular Wg binds to Fz2 predominantly at the basolateral membrane (Strigini and Cohen 2000), suggesting indirectly that canonical Wg/β-cat signaling is initiated at the basolateral cell surface. The existing anti-Fz antibodies are, similarly, not sensitive enough to detect endogenous levels of Fz protein (Krasnow and Adler 1994), but green fluorescent protein (GFP)–tagged Fz (Fz1-GFP) expressed under the control of a ubiquitous promoter shows apical localization in pupal wings and larval eye discs during PCP signaling (Strutt 2001; Strutt et al. 2002). All PCP molecules analyzed (Dsh, Flamingo [Fmi; a.k.a. Starry Night], Strabismus [a.k.a. Van Gogh], Prickle, and Diego ) are also localized in the apical region of pupal wings and eye epithelia (reviewed in Strutt 2003). Importantly, the apical localization of many PCP genes is lost in mutants of Fz1/PCP signaling components, suggesting that Fz/PCP signaling regulates apical localization (Axelrod 2001; Feiguin et al. 2001; Shimada et al. 2001; Strutt 2001; Bastock et al. 2003; Jenny et al. 2003).

Thus, as Fz1 and Fz2 show different subcellular membrane localization within the apical–basal axis, we have here extended our analysis of Fz1 and Fz2 to determine whether the specific subcellular localization is important for signaling readout and to identify the molecular aspects responsible for the localization differences. Our data indicate that localization to apical junction complexes promotes Fz/PCP signaling and inhibits canonical Wg/β-cat signaling, and that the subcellular localization of Fz receptors is mediated through sequences in the cytoplasmic tail (C-tail). In addition, we show that the seven-pass transmembrane region contains elements that are critical for PCP signaling. Based on our data, we propose a model in which subcellular localization, possibly through the association of Fz with other membrane proteins such as coreceptors, is a critical aspect in regulating the signaling readout and specificity within the Wnt/Fz signaling pathways.

## Results

### Different Subcellular Localization of Fz and Fz2 in Imaginal Disc Epithelia

**Figure 1:**
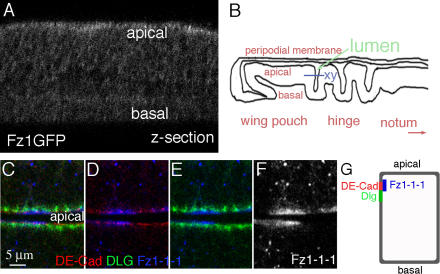
Subcellular Localization of the Fz1 Protein (A) Anti-GFP staining of Fz-GFP in *arm-fz-GFP* third instar wing disc (*arm* drives ubiquitous expression); xz-section is shown. (B) Illustration of a cross section of a third instar wing disc. Wing epithelium forms several folds in the hinge region, where apical–basal localization can be visualized in a horizontal xy-section. The purple line in (B) indicates the position of the xy-optical section in such folds shown in (C–F). (C) Staining of a *dpp-Gal4/UAS-fz1–1-1(myc)* third instar wing disc. Localization of DE-Cad (in red), Dlg (green), and Fz1–1-1 (anti-Myc, blue) is shown. Apical region of the epithelium faces the lumen in the fold, and the basolateral regions are away from the lumen. (D) same staining as in (C) with two channels shown: DE-Cad and Fz1–1-1. DE-Cad (red) and Fz1–1-1 (blue) largely overlap. (E) Dlg (green) and Fz1–1-1 (blue) from (C) are shown. Fz1–1-1 localizes generally more apical than Dlg (with only a very slight overlap). (F) Fz1–1-1 single-channel staining. In summary, Fz1–1-1 is mainly localized in the apical adherens junctions and strong punctae inside cells (probably intracellular vesicles). Low levels of Fz1–1-1 also exist more ubiquitously in the basolateral region. (G) Schematic illustration of relative positions of DE-Cad, Dlg, and Fz1–1-1 along the apical–basal axis epithelial cells. DE-Cad marks the adherens junctions, whereas Dlg localization correlates with septate junctions.

To confirm that Fz1 is localized apically, we analyzed Fz1 distribution in third instar larval discs (Figure 1). Similar to previous reports (Strutt 2001), we found that a ubiquitously expressed Fz1-GFP is always enriched at apical junctions (although the expression in third instar larval discs is weaker than in pupal wings; Figure 1A). Expression of a Myc-tagged Fz1 under *dpp-Gal4* control also displays a strong enrichment in the apical region of the disc epithelium (Figure 1C–1F) and in some punctae that appear to be intracellular vesicles (Figure 1F). There are only low levels of Fz1 detected basolaterally (Figure 1F; unpublished data). Apical Fz1 largely colocalizes with DE-Cadherin (DE-Cad; a marker for adherens junctions; Figure 1D), whereas it only slightly overlaps with Discs large (Dlg) staining (Figure 1E; Dlg is localized to septate junctions just basally to adherens junctions [reviewed in Tepass et al. 2001]). This Fz1 localization pattern is very similar to the PCP factor Strabismus/Vang, which also largely colocalizes with DE-Cad, and only slightly with Dlg (Bellaiche et al. 2004).

Taken together, these data suggest that Fz1 is mostly localized at adherens junctions. This is in contrast to Fz2, which is distributed throughout the cellular membrane along the apical–basal axis in the wing imaginal disc epithelium (Strigini and Cohen 2000). As only Fz1 can signal effectively in the Fz/PCP pathway and other PCP proteins also show apical localization (Strutt 2003), we speculate that apical Fz1 localization is an important feature of signaling specificity.

### The C-Tail of Fz Family Receptors Controls Subcellular Localization

As Fz1 and Fz2 show different subcellular localization, we wished to determine which domains or sequences within the receptors are responsible for the specific localization. To address this question, we examined the localization of Fz1/2 chimeric receptor proteins (expressed under the control of *dpp-Gal4*) in wing imaginal discs. Fz1 and Fz2 were subdivided into three parts: (1) the N-terminal Wnt-interacting cysteine-rich domain (CRD), (2) the remaining proximal extracellular domain and 7 transmembrane region and loop region (collectively referred to as 7-TM), and (3) the intracellular C-tail. All chimeric proteins were Myc-tagged between the CRD and 7-TM region (see Materials and Methods) and labeled with three digits (separated by dashes) corresponding to the three domains of Fz1/2, with “1” and “2” reflecting Fz1 and Fz2 origin, respectively.

**Figure 2:**
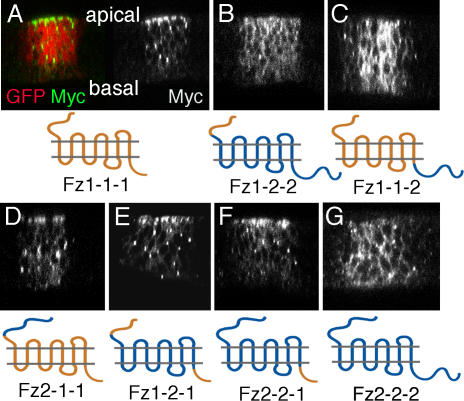
The Cytoplasmic Region of Fz Regulates Subcellular Localization All Fz1/2 chimeras shown are Myc-tagged (the tag being inserted right after the CRD of Fz1 or Fz2; see Materials and Methods; Boutros et al. 2000). The respective Fz1/2 chimeras, with their schematic structure shown under each photomicrograph, were expressed under *dpp-Gal4* (expression domain marked with *UAS-EGFP* in example in [A]) and analyzed by confocal microscopy xz-sections (perpendicular to the stripe of expression in the wing pouch region). (A) Subcellular localization of wild-type Fz-Myc (Fz1–1-1, in green; red channel shows coexpressed GFP to mark expressing cells). Single-channel black-and-white staining of Fz-Myc is shown on right. (B–F) Anti-Myc staining of different Fz1/2 chimeras: (B) Fz1–2-2, (C) Fz1–1-2, (D) Fz2–1-1, (E) Fz1–2-1, and (F) Fz2–2-1. (G) Fz2–2-2. Note the correlation of apical Fz localization with the presence of the Fz1 C-tail.

In all cases tested, the hybrid Fz1/2 proteins carrying the C-tail of Fz1 were enriched apically (Figure 2), comparable to wild-type Fz1, and colocalized with apical junctional markers (see Figure 1; unpublished data). In contrast, chimeric Fz receptors carrying the Fz2 C-tail, including Fz2–2-2, were localized evenly along the apical–basal axis (Figure 2B, 2C, and 2G), comparable to wild-type Fz2 (e.g., endogenous Fz2 [M. Strigini, personal communication] or overexpressed Fz2 under *dpp-Gal4* control [Strigini and Cohen 2000]). In summary, these data indicate that the C-tails of Fz receptors are responsible for their specific subcellular localization.

**Figure 3:**
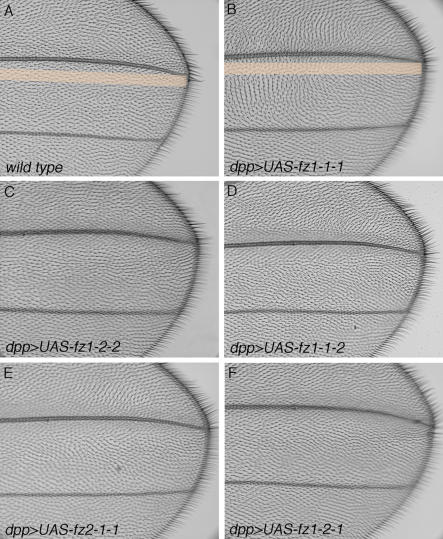
GOF Planar Polarity Wing Phenotype of Fz1/2 Chimeras *dpp-Gal4* was used to express the respective Fz1/2 chimeras in the wing (same as described in Figure 2). (A) Wild-type wing. The *dpp-Gal4* expression domain is highlighted by a thick orange line. In wild-type, all wing hairs are pointing distally. (B) *dpp-Gal4; UAS-EGFP/UAS-fz1–1-1* wing (*dpp>fz1–1-1*; the expression domain is again highlighted with light orange). Wing hairs flanking the expression domain point away from it, consistent with previous observations that hair point away from higher levels of Fz1 activity (Adler et al. 1997). (C) *dpp>fz1–2-2* wing. Wing hairs are not pointing away from expression domain, suggesting that Fz1–2-2 is not active for PCP signaling. (D) *dpp>fz1–1-2* wing. Hairs point away only very slightly (less than 45 ^o^; compare with Fz1–1-1, showing a 90 ^o^ reorientation next to expression domain). Several different lines of *UAS-fz1–1-1* and *UAS-fz1–1-2* were compared, showing identical behavior (Fz1–1-1 having a much stronger phenotype), suggesting that the C-tail is required for full PCP Fz activity. (E) *dpp>fz2–1-1* wing. Most wing hairs point away from expression domain. The phenotype is weaker than Fz1–1-1. (F) *dpp>fz1–2-1* wing. Wing hair orientation is hardly affected. Since Fz1–2-1 is apically localized (see Figure 2E), this result indicates that the presence of the Fz1 7-TM region is important for PCP activity.

To address whether subcellular localization correlates with specific Fz signaling events, we tested the signaling preferences of the respective chimeric receptors. This was analyzed in adult wings by scoring for either a PCP or canonical Wg-signaling gain-of-function (GOF) phenotype (Figure 3; Table 1). Expression of Fz1–1-1 under *dpp-Gal4* control in wing imaginal discs caused wing cell hairs to point away from the expression domain (Figure 3B). This is consistent with the notion that hairs point away from regions of higher Fz signaling levels in the PCP context (Adler et al. 1997). Expression of the chimeric Fz receptors showed that the presence of the Fz1 C-tail is necessary for a strong PCP GOF phenotype (Figure 3; Table 1), suggesting that the apical localization of Fz is important for normal PCP signaling. These experiments also indicated that, in addition to apical localization, the 7-TM region of Fz1 is necessary for effective PCP signaling (Figure 3; Table 1). Similar results were obtained in GOF PCP assays during eye development (Table 1; Boutros et al. 2000; unpublished data).

**Table 1:**
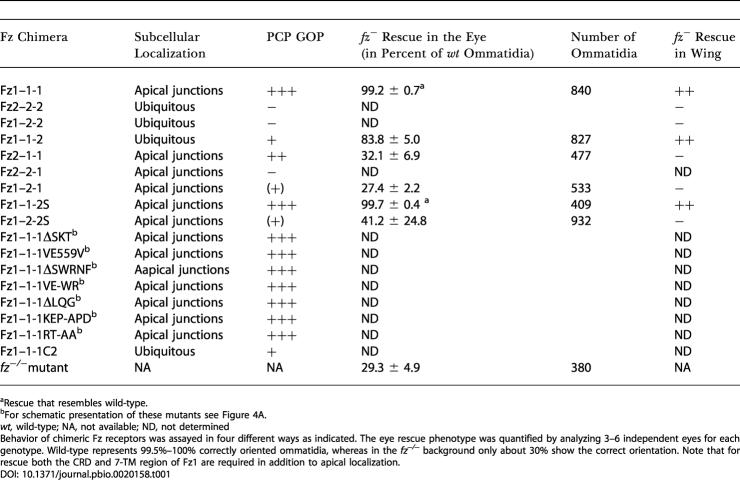
Behavior of Chimeric Fz Receptors

^a^Rescue that resembles wild-type

^b^For schematic presentation of these mutants see Figure 4A

*wt,* wild-type; NA, not available; ND, not determined

Behavior of chimeric Fz receptors was assayed in four different ways as indicated. The eye rescue phenotype was quantified by analyzing 3–6 independent eyes for each genotype. Wild-type represents 99.5%–100% correctly oriented ommatidia, whereas in the *fz^−/−^* background only about 30% show the correct orientation. Note that for rescue both the CRD and 7-TM region of Fz1 are required in addition to apical localization

Taken together, these experiments demonstrate that (1) apical Fz1 localization correlates with higher levels of Fz/PCP signaling activities and (2) the 7-TM region of Fz1 is critical for effective PCP signaling.

### Sequence Requirement for Apical Localization within the C-Tail

**Figure 4:**
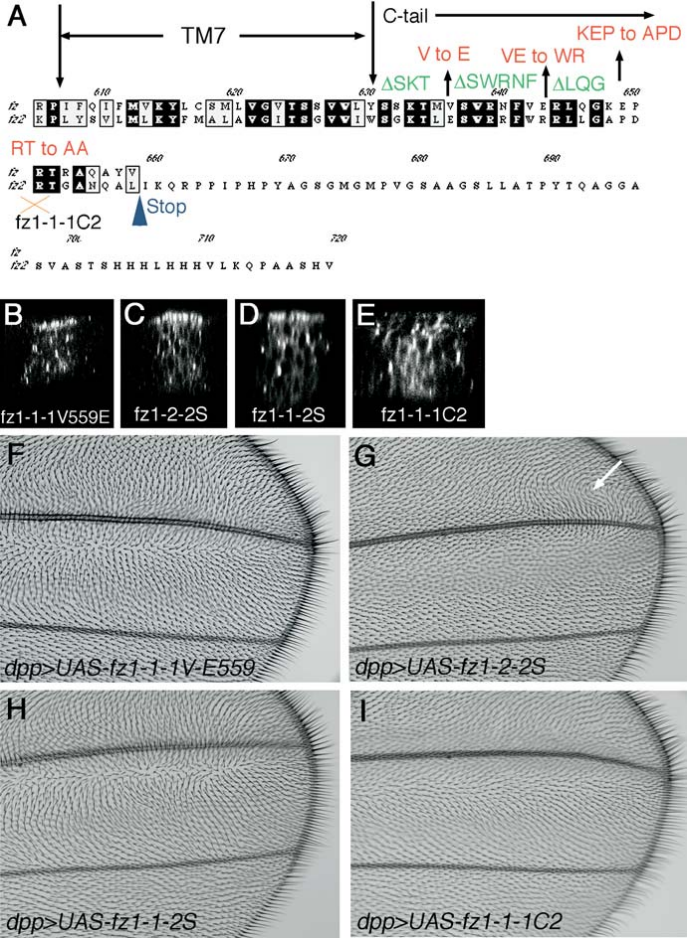
Effects of Fz1/2 C-Tail Mutations on Subcellular Localization and PCP Activity (A) Sequence alignment of Fz1 and Fz2 C-tails. Note high degree of conservation within the membrane proximal shared portion of the Fz1 and Fz2 C-tails. The respective mutations generated and analyzed are indicated above the sequence (see also Table 1 for complete data set). As in Figures 2 and 3, *dpp-Gal4* was used to drive expression of the respective mutants, and these were detected by anti-Myc staining in third instar wing discs. Examples for Fz1–1-1V559E (V to E substitution) are shown in (B) (localization) and (F) (function). All other mutants analyzed as shown in (A) are listed in Table 1. (C–E, G, and H) show the effects of the Fz2 C-tail-specific sequences. The Fz2 C-tail was truncated at the position of the Fz1 stop codon (amino acid L633), yielding a short Fz2 C-tail (2S). The localization (C and D) and GOF PCP function (G and H) of the respective chimeras, Fz1–2-2S and Fz1–1-2S, is shown. Note that both chimeras localize apically (C and D), and Fz1–1-2S shows a strong PCP GOF phenotype (H), very similar to Fz1–1-1 (see Figure 3B). Fz1–2-2S shows only a very weak PCP phenotype (G), mainly occurring at an anterior distal region of the wing (marked by arrow; the rest of the wing is wild-type). (E) Subcellular localization of Fz1–1-1C2. Fz1–1-1C2 is Fz1 with the addition of the Fz2-specific tail extension (see Materials and Methods). Note ubiquitous protein localization within the apical–basal axis (E) and a much reduced PCP activity, as compared to wild-type Fz1–1-1, in the functional assay (I). The phenotype is much weaker than in wild-type Fz1 (compare with [F] and [H] and Figure 3B).

Next we wished to determine which part of the C-tail of Fz1 or Fz2 is responsible for the difference in subcellular localization. The protein sequences of the Fz1 and Fz2 C-tails are homologous over the first 29 amino acids (45% identity), but Fz2 is longer by an additional 61 amino acids (Figure 4). The apical localization sequence could thus be located either in the nonconserved stretches within the common 29 residues, or within the Fz2 C-tail extension. We addressed both possibilities systematically and analyzed the localization of the respective mutants and their effects in the functional GOF assay in the wing (see above).

First, we mutated several Fz1–1-1–specific residues to those of Fz2, or deleted conserved amino acid stretches within the Fz1–1-1 C-tail (see Figure 4A and Table 1 for specific mutations analyzed). All mutated Fz1–1-1 receptor proteins showed normal localization to apical junctions (Figure 4B; Table 1), and when analyzed for their function also showed a typical Fz GOF PCP phenotype in the wing in that the wing hairs were directed away from the source of expression (Figure 4F; Table 1).

Second, we tested whether sequences within the extended Fz2 C-tail have an effect on localization or PCP signaling. We introduced a stop codon after the L633 residue of Fz2 (corresponding to the position of the stop codon in Fz1) in Fz1–1-2 and Fz1–2-2 chimeras (Figure 4A, blue arrowhead), thus truncating the Fz2 C-tail and generating chimeras Fz1–1-2S (“S” for “short”) and Fz1–2-2S. Whereas Fz1–1-2 and Fz1–2-2 are ubiquitously localized, both Fz1–1-2S and Fz1–2-2S localize apically to adherens junctions, in a manner indistinguishable from that of Fz1–1-1 and Fz1–2-1 (compare Figure 4C and 4D to Figure 2A and 2E). These data suggest that the Fz2 C-tail extension interferes with apical localization.

These same chimeras were tested in the functional assay for PCP signaling activity. Strikingly, expression of Fz1–1-2S caused a phenotype very similar to that of Fz1–1-1 (Figure 4H), but different from that caused by Fz1–1-2 (see Figure 3D). Expression of Fz1–2-2S resembled that of Fz1–2-2 or Fz1–2-1, with very weak PCP effects (compare Figure 4G to 3C and 3F). In summary, these results confirm that both apical localization and sequences located within the 7-TM region are functionally important for PCP signaling.

To test whether the extension within the Fz2 C-tail can more generally block apical localization, we added the Fz2 extension on to Fz1–1-1 (Figure 4; see Materials and Methods for details). This Fz1–1-1C2 receptor isoform was not apically enriched (Figure 4E), resembling the localization of Fz1–1-2. Consistently, in the functional PCP readout assay, expression of Fz1–1-1C2 showed only very weak GOF PCP effects (Figure 4I). Based on the results with Fz1–1-1C2 and Fz1–1-2S, we conclude that the Fz2 C-tail extension causes Fz receptors to acquire a ubiquitous membrane distribution, preventing them from accumulating at the apical junctions and thereby affecting their ability to signal via the Fz/ PCP pathway.

### Apical Localization Affects Rescue Capability of the Fz Chimeras

**Figure 5:**
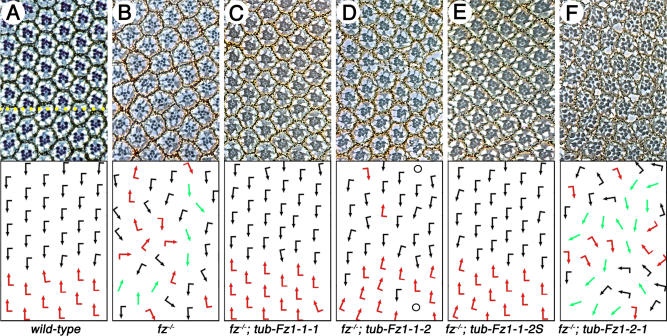
Rescue of the *fz^−^* Eye Phenotype with *tub*-Promoter-Driven Fz Chimeras Tangential eye sections with corresponding schematic in lower part of panel reflecting ommatidial polarity (respective genotypes are also marked below each panel). Black arrows, dorsal chiral form; red arrows, ventral chiral form; green arrows, symmetric ommatidia; black circles, ommatidia with missing photoreceptors. Anterior is to the left, dorsal is up, and an area around the equator is shown for each genotype. (A) Section of a wild-type eye (equator is indicated by yellow line). (B) *fz^P21^/fz^R52^* (*fz* null). Note random orientation of ommatidia. (C) *fz^P21^/fz^R52^*; *tub-fz1–1-1*. The *fz^−^* phenotype is fully rescued (100% with respect to chirality; only a minor rotation wobble is rarely seen). (D) *fz^P21^/fz^R52^*; *tub-fz1–1-2*. Note partial rescue with respect to polarity (approximately 83%) and occasional photoreceptor loss representative of Wg/β-cat signaling. (E) *fz^P21^/fz^R52^*; *tub-fz1–1-2S*. Note 100% rescue, identical to wild-type Fz1 (compare with [C]). (F) *fz^P21^/fz^R52^*; *tub-fz1–2-1*. No rescue due to the presence of the Fz2 7-TM region. This chimera actually shows a mild dominant negative behavior as apparent by the increased percentage of symmetric clusters (approximately 50% as compared to *fz^−^* [approximately 15%]).

The chimeric Fz1/2 receptors (driven directly by the ubiquitous *tubulin promoter [tub]* ) were also tested for their ability to rescue the *fz^−^* eye and wing PCP phenotype. *tub*-Fz1–1-1 and *tub*-Fz1–1-2S (which are both apically localized) fully rescue the *fz^−^* loss-of-function (*fz^P21^*/*fz^R52^*) phenotype in both the eye and wing (Figure 5; Table 1; unpublished data), suggesting that the shortened Fz2 C-tail is functionally equivalent to the Fz1 C-tail. In contrast, *tub*-Fz1–2-2S and *tub*-Fz1–2-1 did not rescue the *fz^−^* mutant phenotype (Figure 5F; Table 1), confirming again that the Fz1 7-TM region is important for Fz/PCP signaling. Although Fz2–1-1 has activity in GOF studies (Figure 3E; Table 1), *tub*-Fz2–1-1 did not rescue the *fz^−^* phenotype, suggesting that the specific extracellular CRD is required for normal receptor regulation (Table 1; unpublished data). This could be due to a Fz1 requirement to interact with a ligand (or extracellular domain of another transmembrane protein) to provide regulation to Fz/PCP signaling.

The *tub-*Fz1–1-2 receptor, which contains the Fz1 7-TM region, but is localized throughout the cellular membrane, is also able to rescue the *fz^−^* eye and wing phenotype. However, it does so less efficiently (Figure 5D; Table 1), and it also causes eye phenotypes reflecting the activation of Wg/β-cat signaling, such as photoreceptor loss (Wg/β-cat signaling during photoreceptor induction and differentiation blocks the development of these cells as photoreceptors; Wehrli and Tomlinson 1998). These effects of Fz1–1-2 suggest that proper apical enrichment is critical for a clean PCP readout, but that a ubiquitously distributed Fz1 chimera might be sufficiently present at apical adherens junctions to allow for partial rescue.

In summary, our data are consistent with the notion that the C-tail provides the information for correct localization required for full and clean PCP signaling specificity, and that sequences within the Fz1 7-TM region and extracellular domain are required for PCP signaling activity or regulation (see also Discussion).

### Flamingo Is Not Required for the Initial Apical Fz Localization

Previous work has shown that Fz1 is not localized to apical junctions in the wings of *fmi* mutants 30–32 h after puparium formation (APF) (Strutt 2001). Similar observations were made in the late third instar eye imaginal disc (Strutt et al. 2002). These data suggest that Fmi is required for apical localization of Fz1 during PCP signaling. Similarly, Fmi depends on Fz1/PCP signaling to maintain its apical junctional localization in wings 30–36 h APF (Usui et al. 1999), suggesting that Fmi and Fz1 localization are interdependent when PCP signaling is active*.* However, this might not reflect initial requirements for apical localization.

**Figure 6:**
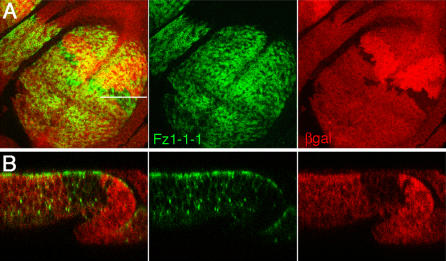
Subcellular Localization of Fz1–1-1 in *fmi^−^* Mutant Clones Fz1–1-1 (Myc-tagged; shown in green) is expressed with *omb-Gal4* (in large parts of the third instar wing pouch). *fmi^E59^* clones were labeled by the absence of anti-βGal staining (red). A projection of several horizontal sections in the apical region (A) and the corresponding xz-section (B) across the clone (as indicated by a white line in A) are shown. Fz1–1-1 is localized apically inside and outside the clone, indicating that initial apical Fz recruitment is independent of Fmi.

To test whether Fmi is required for the initial apical localization of Fz1, which happens prior to the initiation of PCP signaling, we examined Fz1–1-1 localization in *fmi^E59^* clones in larval wing imaginal discs. Fz1–1-1 is localized apically in *fmi^E59^* mutant cells in third instar imaginal discs, indistinguishable from its localization in wild-type tissue (Figure 6). These data suggest that Fmi is not required for the initial apical localization of Fz1–1-1. The difference between the early stage (larval discs) and late stage (pupal wings, late eye discs posterior to morphogenetic furrow during PCP signaling) suggests that initial apical localization is independent of the later maintenance evens regulated by PCP signaling (see also Discussion).

### Apically Localized Fz1/2 Chimeras Act As Dominant Negatives for Wnt/β-Cat Signaling

During imaginal disc development and patterning, Wg binds to the Fz2 receptor at basolateral membranes of the wing epithelium (Strigini and Cohen 2000). This result suggests that canonical Wnt signaling occurs mainly at the basolateral side of the epithelium in imaginal discs. In contrast, apically localized Fz appears to have high PCP signaling activity (as described above). These results suggest that PCP signaling and canonical Wnt/βcat signaling occur in different subcellular locations or membrane compartments.

**Figure 7:**
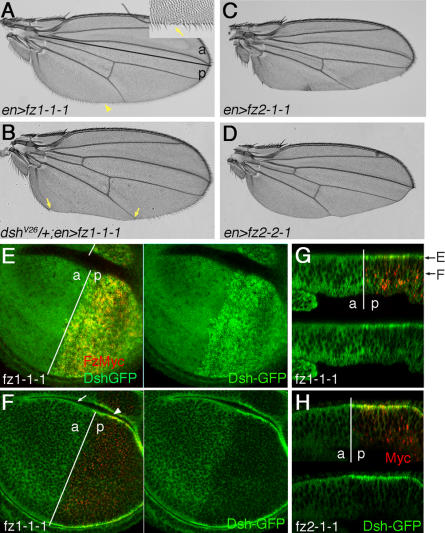
Overexpression of Apically Localizing Fz1/2 Chimeras Has an Inhibitory Effect on Canonical Wnt Signaling (A–D) show adult wings of the respective genotypes. Anterior is up and distal to the right. (A) Adult wing of an *en-Gal4/+; UAS-fz1–1-1/+* fly (*en>fz1–1-1*). *en-Gal4* drives UAS reporter genes only in the posterior compartment. Inset shows high magnification of region marked by arrowhead. Some wing margin bristles are missing (arrow) in the posterior compartment. The border between anterior (“a”) and posterior (“p”) compartments is marked with black line. (B) *dsh^V26^/+; en>fz1–1-1* adult wing. Note enhancement of the margin bristle phenotype: all margin bristles are missing from the area between the arrows in the posterior compartment. (C) *en>fz2–1-1* wing. Most of the wing margin bristles are missing in the posterior compartment. Note also that the posterior compartment is smaller. (D) *en>fz2–2-1* wing. Again the posterior compartment is smaller and most of the margin is missing. (E–G) show that Fz1–1-1 expression increases apical localization of Dsh-GFP and reduces Dsh-GFP in more basolateral areas of wing cells. (E) and (F) are xy-horizontal optical sections, and (G) is an xz-cross section. The positions of (E) and (F) sections are indicated in (G). (E) Apical xy-optical section of a third instar wing disc. Fz1–1-1 (red) is overexpressed by *en-Gal4* in the posterior compartment (anterior–posterior border is labeled by white line, and the corresponding compartments are labeled “a” and “p,” respectively). Dsh-GFP (green) accumulates at higher levels apically in the posterior compartment. Single-channel Dsh-GFP staining is shown at right. In wild-type disc, Dsh-GFP is evenly distributed with no anterior–posterior bias (not shown). (F) A more basal xy-section of the same disc as in (E). Note reduction of Dsh-GFP staining in the posterior compartment, except at the apical junctions as seen in folds (arrowhead). In the anterior compartment, where Fz1–1-1 is not overexpressed, Dsh-GFP is only slightly enriched in the apical folds (arrow). (G) xz-section of the same wing disc shown in (E) and (F), with top panel showing double labeling for anti-Myc (red) and anti-Dsh-GFP (green) and bottom panel showing single channel of Dsh-GFP staining. (H) xz-section of a comparable disc expressing Fz2–1-1 in the posterior compartment*.* Fz2–1-1 overexpression (red) also causes accumulation of Dsh-GFP in apical junctions and reduction of Dsh-GFP along the basolateral region.

Previous work has suggested that Fz2–1-1 and Fz2–2-1, which are shown here as localized to apical junction complexes (see Figure 2D and 2F), act as dominant negative isoforms for canonical Wg signaling (Boutros et al. 2000). We have noticed that expression of the Fz chimeras (with *en-Gal4* in the posterior wing compartment) often causes wing notching and loss of wing margin bristles (in the posterior wing region; Figure 7), indicative of reduced Wnt/βcat signaling (Couso et al. 1994). To gain insight into why chimeric Fz receptors can behave as dominant negatives, we analyzed ubiquitous Dsh-GFP localization (expressed from the endogenous promoter; Axelrod 2001) in *en-Gal4-* and *dpp-Gal4-*driven *UAS-fz* wing discs (Figure 7; unpublished data). In wild-type, Dsh-GFP is mainly cytoplasmic with a mild, slightly stronger apical enrichment at membranes (Figure 7E–7H, anterior compartments). In wing epithelia with overexpressed Fz1–1-1 or Fz2–1-1, much more Dsh-GFP is recruited apically in cells expressing the Fz chimeras (Figure 7E–7H, posterior compartments). At the same time, Dsh-GFP levels are reduced in basolateral regions of these cells. These data suggest that Fz in adherens junctions (apical) is trapping Dsh there, depleting it away from Wnt/βcat signaling components located possibly more basally and thus reducing canonical Wnt signaling.

To test this hypothesis, we analyzed the effect of reducing *dsh* gene dosage in *en-Gal4/UAS-fz1–1-1* flies, where wing notching and loss of marginal hairs is mild (21% of wings have large areas of margin bristles missing; Figure 7A; Table 2). Strikingly, the *en-Gal4/UAS-fz1–1-1* effect is enhanced in *dsh* heterozygous flies (*dsh^V26^*/+), with 65% of wings showing large areas of margin bristles missing and severe wing notching (Table 2; see Figure 7B for example). To corroborate the *dsh* dosage sensitivity in this context, we generated flies with three copies of *dsh* (by introducing an additional *dsh* copy as a *dsh-GFP* transgene expressed under its endogenous promoter; Axelrod 2001). In this genetic background with three *dsh* copies, only 4% of the *en-Gal4/UAS-fz1–1-1* wings displayed a large area of missing wing margin bristles (Table 2), suggesting that the presence of extra Dsh suppresses the *en-Gal4/UAS-fz1–1-1* wing phenotype. Taken together, these Dsh dosage effects support the idea that trapping Dsh into apical junctional complexes reduces its availability for Wnt/βcat signaling, and thus reduces the strength of canonical signaling.

**Table 2:**
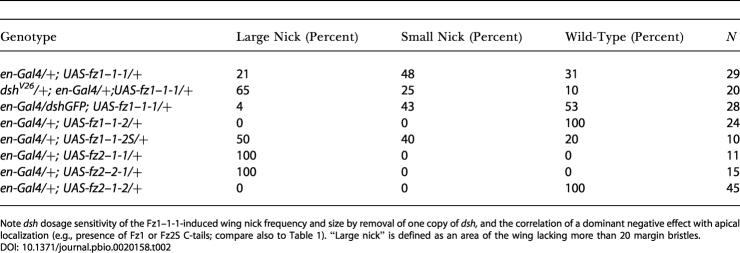
Wing Margin Phenotypes of *en-Gal4; UAS-fz1/2* Chimeras

Note *dsh* dosage sensitivity of the Fz1–1-1-induced wing nick frequency and size by removal of one copy of *dsh,* and the correlation of a dominant negative effect with apical localization (e.g., presence of Fz1 or Fz2S C-tails; compare also to Table 1). “Large nick” is defined as an area of the wing lacking more than 20 margin bristles

In further support of this explanation, *en-Gal4/+; UAS-fz1–1-2/+* flies show only a very mild effect on wing margin bristles (Table 2). As Fz1–1-2 is ubiquitously localized along the apical–basal axis, recruiting of Dsh by such chimeras should not have an adverse effect on canonical Wg signaling. In contrast, when Fz2–1-1 and Fz2–2-1 are expressed (with *en-Gal4*) we observe very strong wing notching effects and a general reduction of the posterior wing compartment (Figure 7C and 7D; Table 2). This can be explained as follows. As the Fz2 ligand-binding CRD has a much higher affinity for Wg than the Fz1 CRD (Rulifson et al. 2000), the strong dominant negative behavior of Fz2–1-1 and Fz2–2-1 can be explained by adverse effects on both Dsh and Wg: Fz2–1-1 and Fz2–2-1 have a high-affinity Wg-binding CRD (sequestering Wg efficiently) and can trap Dsh at junctional complexes as well (Figure 7E–7H), making large pools of Wg and Dsh unavailable for canonical signaling, and thus causing a strong dominant negative effect.

In summary, the dominant negative effect of the overexpression of Fz1–1-1, Fz2–1-1, and Fz2–2-1 is caused by trapping Dsh into apical junctions, making it unavailable for canonical Wnt/βcat signaling, and, when present, the Fz2 CRD enhances this effect by also sequestering Wg to these complexes. These results suggest that a Fz-Dsh complex in the apical junctions is largely incapable of canonical β-cat signaling, suggesting that the subcellular localization of Fz receptors contributes significantly to the signaling outcome and specificity (see Discussion).

## Discussion

We have shown that Fz1 and Fz2 have different subcellular localizations within the wing imaginal epithelium. This difference is mediated by sequences in the cytoplasmic tail of Fz2 that appear to block apical accumulation. The subcellular localization difference directly contributes to the signaling specificity outcome. Whereas apical localization favors Fz/PCP signaling, it interferes with canonical Wnt/β-cat signaling.

### The Relationship between Apical Localization of Fz1 and Its PCP Signaling Activity

Is the apical localization of Fz required for PCP signaling? The Fz1–1-2 chimera, which is distributed ubiquitously within the apical–basolateral membrane, only partially rescues the *fz^−^* eye phenotype, and it can also cause defects related to canonical Wg/Arm signaling (see Figure 5D). In contrast, apically localized Fz1–1-2S fully rescues the *fz^−^* phenotype and has no additional effects. The Fz1–1-2 chimera also shows much weaker PCP phenotypes in the GOF assay (see Figure 3 and Boutros et al. [2000]). Taken together, these results suggest that a reduction in the apical localization of Fz leads to a reduction in PCP signaling activity. However, about 80% of the chirality defects in *fz^−^* eyes are rescued by *tub-fz1–1-2*, and in the wing *tub-fz1–1-2* rescues the *fz^−^* mutant to a similar extent as *tub-fz1–1-1* and *tub-fz1–1-2S* (unpublished data), suggesting that Fz1–1-2 contains substantial PCP signaling activity.

Because both GOF and loss-of-function studies indicate that the Fz1 7-TM region is critical for Fz1 function, Fz1–1-2 is expected to have Fz/PCP signaling activity, although with altered subcellular distribution. Thus, the remaining PCP signaling activity of Fz1–1-2 seen is probably due to the presence of some of this protein in apical regions. It is difficult to determine how much of Fz1–1-2 is actually localized to this membrane region. Since the immunohistochemical staining indicates that it is not excluded apically, we assume that Fz1–1-2 has enough apical localization to participate when PCP signaling is initiated. It has been suggested that wing cell orientation does not depend on absolute Fz levels, but instead depends on relative Fz/PCP activity differences in a Fz activity gradient across a field (Adler et al. 1997). Thus, although the absolute activity of Fz1–1-2 is reduced (based on weaker GOF phenotypes and weaker rescue of *fz^−^* in the eye), the relative difference might be sufficient for the partial rescue.

In this context, it is worth noting that *tub-fz1–1-2* rescues the *fz^−^* phenotype better in the wing than in the eye, whereas there is no apparent difference in rescue activity between the eye and the wing for *tub-fz1–1-1* or *tub-fz1–1-2S*. The difference could be due to the observed nonautonomous PCP signaling effects in the wing (Vinson and Adler 1987), where neighboring cells affect each other's planar polarization. Fz1–1-2 may allow some wing cells to adopt the correct orientation, which then in turn influences many of the remaining wing cells to also orient themselves correctly through nonautonomous interactions.

### Regulation of Fz Apical Localization

It has been shown that Fz1 localization is affected in *fmi* mutant clones at about 30 h APF (Strutt 2001), leading to the proposal that Fmi recruits Fz1 into apical junctions (Strutt 2001; Bastock et al. 2003). However, we find that Fz1 is localized normally in *fmi* null mutant clones earlier in the third instar wing disc. What causes the difference between these two observations?

PCP signaling in the wing is thought to act in two phases (one 6–24 h APF and the second 24–32 h APF [Strutt and Strutt 2002]), and it results in the distal enrichment and maintenance of Fz1 (Strutt 2001). As Fz1/PCP signaling is modulated by Fmi (Usui et al. 1999), Fmi-dependent changes in Fz1 localization likely result from effects on PCP signaling activity. At the same time, Fmi localization is also dependent on Fz1 activity and becomes also less apically localized in *fz^−^* tissue at 30–36 h APF (Usui et al. 1999), suggesting that the regulation of apical localization between Fz1 and Fmi is complicated and mutual at these late stages.

We showed here that initial apical localization of Fz1, preceding both stages of PCP signaling, is not *fmi* dependent. This result suggests that Fmi and Fz1 get recruited to apical junctions independently. During later stages, Fmi and Fz1 then affect each other's localization through PCP signaling. At this point, it remains unclear which molecules initially recruit Fz1 into the apical junctional region.

### Fz Receptor Localization and Canonical Wnt Signaling

Secreted Wg mainly binds to Fz2 at basolateral membrane regions of the wing epithelium (Strigini and Cohen 2000), indirectly suggesting that canonical signaling occurs in the basolateral membrane compartment. Our experiments show that overexpression of Fz1–1-1 or Fz2–1-1 leads to a cell-autonomous loss of wing margin bristles and associated tissue, suggesting that these molecules act like dominant negatives, inhibiting Wnt/β-cat signaling. As these molecules are enriched apically and sequester Dsh there, Fz-Dsh complexes at apical junctions may be largely inactive for canonical Wnt signaling. This result suggests that canonical Wnt signaling and PCP signaling occur in different subcellular compartments. Basolateral Wnt/β-cat signaling is also suggested by the fact that (1) secreted Wg binds to Fz2 at the basolateral membrane and that (2) apical Wg secretion and signaling could lead to mis-specification in disc folds and cells in the peripodial membrane (Strigini and Cohen 2000).

Both Fz1 and Fz2 are capable of canonical Wnt/β-cat signaling (Bhat 1998; Kennerdell and Carthew 1998; Bhanot et al. 1999; Chen and Struhl 1999). Consistently, different Fz1/2 chimeras, including related versions of Fz2–1-1 and Fz2–2-1, are capable of rescuing the *fz*, *fz2* double mutant phenotype (Strapps and Tomlinson 2001). However, when Fz1–1-1, Fz2–1-1, or Fz2–2-1 is expressed at high levels, Dsh accumulates at apical junctions, thus decreasing cytosolic Dsh levels. As the chimeric receptors can rescue the *fz, fz2* double mutant when expressed at low levels (under the control of the *tub* promoter; Strapps and Tomlinson 2001), the relative level of each receptor together with its subcellular localization appear critical for the signaling outcome.

In conclusion, we have shown that subcellular localization contributes to Fz signaling specificity. Our data indicate that the localization of Fz1 at apical junctions promotes Fz/PCP signaling, whereas this localization can inhibit canonical Wnt/β-cat signaling. The localization is mediated through sequences in the C-tail.

## Materials and Methods

### 

#### Flies and constructs

The flies carrying the chimeric receptor constructs UAS-Fz1–1-1, UAS-Fz1–1-2, UAS-Fz1–2-2, UAS-Fz2–1-1, and UAS-Fz2–2-1 are described in Boutros et al. (2002). UAS-Fz1–2-1 was constructed by combining the Fz1 CRD (Fz1 residues 1–166) with the Myc tag, the Fz2 7-TM region (amino acids 220–617), and the Fz1 C-tail (amino acids 558–585). A HindIII site (generated in vitro) was used to combine Fz1 CRD with the Fz27-TM region. An XhoI site was used to link the Fz2 7-TM region with the Fz1 C-tail. C-tail mutation constructs of Fz1 were generated through PCR-based site-directed mutagenesis (Quikchange kit, Stratagene, La Jolla, California, United States). Fz1–1-2S and Fz1–2-2S were generated by introducing a stop codon after residue L633 of Fz2. Fz1–1-1C2 was generated by introducing a BsiWI site at the residues R574–T575 of Fz1 and R626–T627 of Fz2 (the RT residues remain the same by this mutagenesis). This added the Fz2 amino acids 627–694 to the Fz1 C-tail at the RT residues. The respective UAS transgenic flies were generated by standard procedures.

The Gal4/UAS system was used to express the chimeric UAS-Fz1/2 transgenes (Brand and Perrimon 1993) with *dpp-Gal4, en-Gal4,* or *omb-Gal4* (Brand and Perrimon 1993; Yoffe et al. 1995; Lecuit et al. 1996; Morimura et al. 1996). *tub*-promoter-driven Fz chimeric constructs were generated by cloning the respective Fz1/2 constructs into the *Casper4-tub* vector (containing a 2.4-kb *tub* promoter fragment in *Casper4—*a kind gift from Stephen Cohen). *fz^P21^* and *fz^R52^* are null alleles of *fz* (Jones et al. 1996). *dsh^V26^* is a null allele of *dsh* (Perrimon and Mahowald 1987).

#### Immunohistochemistry

Rat anti-DE-Cad was used at 1:200 (Oda et al. 1994). Mouse anti-Myc (9E10) was used at 1:250–500 (Santa Cruz Biotechnology, Santa Cruz, California, United States). Rabbit anti-Dlg was used at 1:3500 (Lee et al. 2003). Rabbit anti-GFP (Molecular Probes, Eugene, Oregon, United States) was used at 1:4000 to detect Dsh-GFP and Fz1-GFP.

*fmi^E59^* clones were induced in first instar larvae via the Flp/FRT system in the *w, hs-flp; FRT42B fmi^E59^/FRT42 arm-lacZ* genotype*.* Larvae were dissected 4 d after clone induction during late third instar. *fmi^E59^* is a null allele of *fmi* (Usui et al. 1999).

#### Adult wing and eye preparation

Wings were soaked (with agitation) in 0.1% Triton X-100 PBS for about 30 min or longer, and then mounted in 80% Glycerol PBS. Eye embedding and sectioning was performed as described by Tomlinson (1987).

## Abbreviations

7-TM: proximal extracellular domain and 7 transmembrane region and loop region
β-cat: β-catenin
APF: after puparium formation
Arm: Armadillo
CRD: cysteine-rich domain
C-tail: cytoplasmic tail
DE-Cad: DE-Cadherin
Dlg: Disks large
Dsh: Disheveled
Fmi: Flamingo
Fz: Frizzled
GFP: green fluorescent protein
GOF: gain-of-function
PCP: planar cell polarity
*tub*: *tubulin*
